# Nutritional Deficiency in Patients with Heart Failure

**DOI:** 10.3390/nu8070442

**Published:** 2016-07-22

**Authors:** Edoardo Sciatti, Carlo Lombardi, Alice Ravera, Enrico Vizzardi, Ivano Bonadei, Valentina Carubelli, Elio Gorga, Marco Metra

**Affiliations:** Cardiology, Department of Medical and Surgical Specialties, Radiological Sciences and Public Health, University of Brescia, Piazzale Spedali Civili 1, Brescia 25123, Italy; edoardo.sc@tin.it (E.S.); ravera.alice@gmail.com (A.R.); enrico.vizzardi@tin.it (E.V.); ivano.bonadei@libero.it (I.B.); valentina.carubelli@gmail.com (V.C.); eliogorga@yahoo.it (E.G.); metramarco@libero.it (M.M.)

**Keywords:** heart failure, nutritional deficiency, metabolism, iron, amino acids, coenzyme Q10, cardiac cachexia

## Abstract

Heart failure (HF) is the main cause of mortality and morbidity in Western countries. Although evidence-based treatments have substantially improved outcomes, prognosis remains poor with high costs for health care systems. In patients with HF, poor dietary behaviors are associated with unsatisfactory quality of life and adverse outcome. The HF guidelines have not recommended a specific nutritional strategy. Despite the role of micronutrient deficiency it has been extensively studied, and data about the efficacy of supplementation therapy in HF are not supported by large randomized trials and there is limited evidence regarding the outcomes. The aim of the present review is to analyze the state-of-the-art of nutritional deficiencies in HF, focusing on the physiological role and the prognostic impact of micronutrient supplementation.

## 1. Introduction

Heart failure (HF) is the main cause of mortality and morbidity in Western countries [1]. Acute HF (AHF) is the leading cause of hospitalization in the Unites States and Europe. HF patients have multiple comorbidities such as renal dysfunction, pulmonary disease, anemia and depression, which influence the prognosis [2]. Despite the introduction over the last 20 years of renin-angiotensin-aldosterone (RAA) system antagonists, beta-blockers and devices have improved the outcomes, the quality of life and the functional capacity remains extremely poor, especially in the advanced stages of the disease [1,3]. The mortality and hospitalization rates remain unacceptably high also due to lack of effective new therapies in AHF [4,5].

The unsatisfactory quality of life emphasizes depression, discontinuation of therapy, and insufficient intake of nutrients [6,7,8]. In patients with HF, the increased risk of poor dietary intake is associated with lower quality of life, which predicts cardiovascular events [9,10]. However, the HF guidelines have not recommended specific nutritional strategies, despite growing evidence regarding the important role of micronutrient deficiencies in chronic HF [1,11]. Clinical research investigating the effects of micronutrient supplementations on the outcomes is still limited. The lack of general recognized nutritional strategies aimed at improving the quality of life and functional capacity remains an unmet need in HF research.

The aim of the present review is to analyze the state-of-the-art of nutritional deficiency in HF, focusing on the evidence regarding the physiological role and the prognostic impact of supplementation therapies in HF patients.

## 2. Metabolism Deficiencies in Heart Failure

The failing heart is an energy-compromised organ, characterized by “metabolic remodeling” [12]. Abnormalities in myocardial substrate utilization and energy metabolism, including a decline in adenosine triphosphate (ATP), mitochondrial dysfunction, and the increase in free fatty acid concentration, are directly correlated with the progression of HF [13,14,15,16]. The shift toward carbohydrate utilization from fatty acids (FAs) oxidation represents a fetal metabolic phenotype activated by the expression of specific fetal genes [17,18]. The over-expression of fetal genes reduced the cardiac efficiency by converting chemical energy into mechanical work [19,20]. In addition, ischemia and oxidative stress may further reduce energy expenditure, resulting in cardiomyocyte injury [21,22].

Several micronutrients are essential cofactors of metabolic reactions and contribute to the efficiency and the appropriate utilization of energy [23]. The genetic deficiencies of l-carnitine, thiamine, and taurine are associated with specific cardiomyopathies [24]. It has also been demonstrated that the failing heart is deficient in several micronutrients [25]. Consequently, the administration of micronutrients should be an attractive option for HF patients.

HF is characterized by loss of muscle mass and function (sarcopenia), and/or loss of tissue mass (cachexia) [26,27]. It has been demonstrated that unintentional weight loss is an independent risk factor for mortality in chronic HF [28,29]. Cardiac cachexia is characterized by a catabolic state in which micronutrient supplementation may play a crucial role [30]. Despite this evidence, the efficacy of nutritional intervention is less clear. In the paragraphs below, we analyze the state-of-the-art knowledge about single micronutrient supplementation in HF.

The effects of supplementation in HF patients are reported in Table 1 and Table 2, respectively.

## 3. Coenzyme Q10

Coenzyme Q10 (CoQ10) is a natural antioxidant synthesized and diet-supplied lipid-soluble cofactor that acts in the mitochondrial membrane [38]. CoQ10 is present in high concentrations in the mitochondrial electron transport chain (ETC) from complex 1 (NADH coenzyme Q reductase) to complex 3 (cytochrome bc1 complex), and from complex 2 (succinate dehydrogenase) to complex 3. CoQ10 participates in the synthesis of ATP and in healthy subjects is present in high concentrations in the myocardium [39]. It exerts three main biological roles in humans: contributes to mitochondrial energy production, stabilizes the cell membrane, and has an antioxidant effect [40]. Coenzyme Q10 deficiency is associated with HF. In patients with HF, the reduced intake of CoQ10 is correlated with New York Heart Association (NYHA) functional class, lower left ventricular ejection fraction (LVEF) and increased NT-proBNP levels [32]. In addition, beta-blockers and statins have been proved to reduce CoQ10 plasmatic concentration [41,42,43]. The administration of CoQ10 on left ventricular function (i.e., LVEF and cardiac output) has been analyzed in several small studies and some double-blinded clinical trials, suggesting a positive role of CoQ10 supplementation evidenced by an improvement (3.7%) in LVEF [44,45]. Sub-group analysis highlighted the major benefit in lower NYHA classes, LVEF < 30% and patients not assuming ACEi. Antioxidant effects of CoQ10 may prevent myocytes injury and lipid oxidation due to reactive oxygen species. CoQ10 preserves nitric oxygen release, reducing the peripheral vascular resistances (i.e., afterload) and protecting the myocardium against ischemia [46]. Other studies have evaluated and confirmed the positive role of CoQ10 in symptom relief, 6 min walking distance (6MWD), exercise duration, peak oxygen consumption and quality of life [47,48,49].

Observational studies have demonstrated that the serum CoQ10 concentration is an independent predictor of mortality in patients hospitalized for HF, which has not been confirmed in a larger cohort after adjustment for several variables [30,31]. These studies were limited by the small number of patients enrolled and the heterogeneity of clinical endpoints. From 2003 to 2010, 17 centers worldwide conducted the first prospective, randomized, double-blind, placebo-controlled Q-SYMBIO trial regarding CoQ10 as an adjunctive treatment for chronic HF ((100 mg three times daily) vs. placebo (*n* = 420)) [35]. The primary short-term (16 weeks) endpoints were NYHA functional class, 6MWD, and NT-proBNP, while the primary long-term (106 weeks) endpoint was composite major adverse cardiovascular events (MACE, i.e., hospitalization for worsening HF, cardiovascular death, mechanical assist implantation, or urgent cardiac transplantation). CoQ10 added to standard therapy did not affect the short-term endpoint, but significantly reduced the composite endpoints of MACE (15% vs. 26%, corresponding to a 43% relative reduction) as well as cardiovascular death, hospitalization for HF, and all-cause death (42% relative reduction). However, despite the high number of studies showing favorable effects of CoQ10 supplementation, a recent systematic review has underlined the frailty of the evidence in HF patients [50]. The authors analyzed seven studies in which coenzyme Q10 was compared with a placebo. Despite minimal improvements in the LVEF values and symptoms, the improvement in outcomes, functional capacity, and brain natriuretic peptide values (BNP) were not confirmed. Larger randomized studies are warranted in order to clarify previous conflicting results.

## 4. Vitamin D

In patients with HF, intracellular ionized calcium (Ca^2+^) utilization is impaired and myocardial contractility is negatively affected [51]. Vitamin D plays an important role in bone mineralization and maintenance of the serum calcium level and its receptors were identified in cardiomyocytes and endothelial cells [52,53]. In animal models with vitamin D deficiency, the administration of calcitriol has improved heart contractility and reduced the natriuretic peptides concentration [54,55]. Moreover, vitamin D suppresses renin production, modulates myocardial and vascular hypertrophy, inhibits inflammatory cytokines, improves endothelial function and reduces atherosclerosis [56,57,58,59]. Observational studies have demonstrated that vitamin D deficiency and hyperparathyroidism are common in HF, regardless of age and renal function [60]. In patients with HF renal dysfunction is the most common comorbidity which influences the prognosis and it also represents the main cause of vitamin D deficiency [61,62]. Diuretic therapy, insufficient ultraviolet exposure, inadequate intake with diet and/or malabsorption caused by intestinal edema reduce the vitamin D concentration [60]. In addition, TNF-α, whose plasmatic levels are higher in HF, abolishes calcitriol synthesis, reducing vitamin D levels [63,64].

Liu et al. investigated the prognostic role of low vitamin D status in 548 HF patients with reduced LVEF [33]. After multivariable adjustment, they demonstrated that 25OH-vitamin D concentration independently predicts the combined endpoint of all-cause mortality and HF hospitalization (HR 1.09 per 10 nmol/L decreased; 51% vs. 64% survival in first and third tertile, respectively) and all-cause mortality alone (HR 1.10; 65% vs. 79% survival in first and third tertile, respectively) at 18-month follow-up. In this study, low vitamin D levels were associated with an increase in plasmatic renin activity and C-reactive protein, and even LVEF was inversely related to vitamin D concentration as demonstrated in previous research [65,66,67].

Three recent randomized, double-blind, placebo-controlled trials investigated the effect of vitamin D repletion on functional capacity (i.e., cardiopulmonary stress testing, 6MWD, isokinetic muscle testing) in HF patients with vitamin D deficiency. The results did not show an improvement in physical performance, despite a significant increase in plasmatic vitamin D levels and a reduction in aldosterone concentration [68,69,70]. A randomized trial is ongoing to investigate the impact of long-term vitamin D daily administration regarding 6MWD, left ventricular function at cardiac magnetic resonance, cardiopulmonary exercise testing and biochemical changes (VitamIN D treating patIents with Chronic heArT failurE, VINDICATE, NCT01619891). Another ongoing randomized double-blind placebo-controlled trial is recruiting HF patients with low vitamin D levels to demonstrate whether its replacement could improve long-term prognosis when added to standard of care therapy (Vitamin D and Mortality in Heart Failure, EVITA, NCT01326650).

In conclusion, vitamin D supplementation may be a good target to improve frailty, fatigue and functional decline in patients with HF, but more data are necessary to recommend it for HF patients.

## 5. Iron

Iron is a key element in the human metabolism, it is involved as an enzyme in oxygen transport and storage, and it is involved in the oxidative metabolism in the heart and skeletal muscles [71]. Iron deficiency is the most common nutritional disorder, affecting one-third of the general population all over the world and determining premature death [72,73,74]. Iron deficiency contributes to cardiac and peripheral dysfunction and is associated with increased risk of death, independent of the hemoglobin level [75]. Iron deficiency affects nearly 40% of HF patients and it is associated with a proinflammatory state which impairs the intestinal absorption of iron itself [71]. In HF patients, iron deficiency can be defined as ferritin <100 mg/L (absolute iron deficiency, related to depletion of iron stores), or 100–300 mg/L with transferrin saturation <20% (functional iron deficiency, related to systemic inflammation). Jankowska et al. demonstrated that iron deficiency is a strong and independent predictor of prognosis in HF, with a 1.58 increased risk of death or heart transplant, suggesting a possible therapeutic role of iron implementation [34]. Indeed, several trials have demonstrated that repletion of iron deficiency improves symptoms, exercise capacity and quality of life in HF patients [76,77,78,79]. The EFFECT-HF trial (Effect of Ferric Carboxymaltose on Exercise Capacity in Patients with Iron Deficiency and Chronic Heart Failure) is currently recruiting patients to analyze the role of ferric carboxymaltose on peak VO2 (NCT01394562). Recently, the CONFIRM-HF trial (Double-Blind, Randomized, Placebo-Controlled Study to Assess the Effects of Intravenous Ferric Carboxymaltose on Functional Capacity in Patients with Chronic Heart Failure and Iron Deficiency) demonstrated a 61% relative decrease in HF hospitalization for worsening HF and an improvement in functional capacity, symptoms, and quality of life in patients receiving intravenous ferric carboxymaltose [80]. A meta-analysis of five randomized clinical trials suggested that intravenous iron supplementation reduces cardiovascular hospitalization (OR 0.44), cardiovascular death or hospitalization for worsening HF (OR 0.39), while improving quality of life, functional capacity and other symptoms [36]. On the other hand, excessive iron supplementation may lead to tissue deposition and to free radical tissue damage, so mortality-driven studies about intravenous iron are still needed to assess long-term safety. However, the current European Society of Cardiology (ESC) guidelines on the management of HF recommend searching for reversible causes of HF and comorbidities and suggest that iron therapy can be considered as a treatment in patients with iron deficiency [1].

## 6. Thiamine

Thiamine is one of the first compounds recognized as a vitamin, known as vitamin B1. Thiamine acts as a coenzyme for oxidation-reduction reactions in the glucose metabolism and in the citric acid cycle, and as a coenzyme in the reactions catalyzed by the enzyme pyruvate dehydrogenase [81]. The active form thiamine pyrophosphate (TPP) is involved in bioenergetics ATP synthesis processes, hemoglobin production, γ-aminobutyric acid (GABA) synthesis, immunity stress response, and gene expression [82]. Thiamine deficiency can be associated with alcoholism, hemodialysis, poor nutrition, cancer, pregnancy, bariatric surgery, and Alzheimer’s disease [83]. Beriberi syndrome is a disease caused by a vitamin B1 (thiamine) deficiency [84]. There are two types of this syndrome: wet beriberi and dry beriberi. Wet beriberi affects the heart and circulatory system. Dry beriberi damages the nerves and can lead to a loss of muscle strength and finally muscle paralysis. When the cardiovascular involvement is predominant (“wet” beriberi), patients present biventricular dysfunction, vasodilatation, tachycardia and fluid retention [85]. In Western countries this syndrome is quite rare and mainly related to alcoholism and malnutrition and/or malabsorption. However, a subclinical thiamine deficiency is common in HF patients with long-term furosemide intake, elevated basal metabolic rate, malnutrition and advanced age [86,87]. According to Hanninen et al., about one-third of hospitalized patients affected by chronic HF showed a thiamine deficiency and even small doses of supplementation (1.5 mg/day) may be effective in reducing this deficiency [88]. Only two small randomized placebo-controlled trials demonstrated that thiamin administration in HF patients improves LVEF, diuresis, natriuresis and quality of life [89,90].

A randomized trial conducted in patients with AHF did not show an improvement in dyspnea 4 h after thiamine administration, or on hospitalization rate and duration of hospital stay [91]. Two recent systematic reviews and meta-analysis confirmed the beneficial effect on LVEF [92,93].

Nevertheless, no studies to date were considerable enough to prove a prognostic role of thiamine deficiency correction in HF. In conclusion, thiamine supplementation has beneficial effects on cardiac function in HF patients, but larger studies are needed to test its prognostic significance.

## 7. Creatine

Creatine is an important energetic molecule in skeletal muscles and the heart; it is able to store and transfer high-energy phosphate. Creatine is synthesized from arginine, glycine, and methionine in the kidneys, liver, and pancreas [94]. In healthy people, the dietary supplement of creatine (about 20 g/day for five days or about 2 g/day for 30 days) results in increased skeletal muscle creatine and phosphocreatine [95]. In HF its concentration is reduced, probably as a consequence of increased sympathetic activity [96]. Gordon et al. demonstrated that oral supplementation of 20 g creatine daily for 10 days in 17 patients with chronic HF did not improve LVEF but increased skeletal muscle energy-rich phosphagens and performance as regards both strength and endurance [97]. Few studies have evaluated the role of its supplementation in HF patients, demonstrating possible beneficial effects regarding exercise capacity, muscle strength and metabolism [98,99,100]. No data are available about its prognostic significance if added to standard-of-care therapy.

## 8. Amino Acids

Amino acids (AAs) are fundamental compounds of proteins and their metabolism represents a complex process involving a large number of fundamental metabolites, which are found in vitamins, dietary proteins and nutritional supplements [101]. AAs’ availability is reduced in HF and such patients develop a hypercatabolic state that results in cardiac cachexia [102,103]. HF patients’ AAs are highly metabolized by skeletal muscles even at rest [104]. During the hypercatabolic state, muscle proteins are degraded and AAs are released, leading to a progressive loss in muscle volume. AA deficiency changes the cardiac structure with a transition from the “red fibers” that are more efficient in energy utilization to the “white fibers” that are less efficient [105]. Mixed AA administration has been demonstrated to improve myocardial performance and it stimulates protein synthesis itself [106,107]. Several studies have evaluated the effects of AA single supplementation in HF patients.

### 8.1. Taurine

Taurine is a semi-essential non-codified AA, resulting from cysteic acid decarboxylation. It is expressed at high levels in the myocytes, but its function has not been completely clarified yet [108]. Taurine has antioxidant properties, and regulates the phosphorylation of two myocardial key proteins in excitation-contraction coupling, phospholamban and sarcoplasmatic reticular Ca2+ ATPase (SERCA2), and it also contributes to the function of mitochondria enzymes [109,110,111]. The taurine-deficient heart has an impaired aerobic metabolism and its deficiency is associated with a reduction in oxygen consumption, an elevation in glycolysis and lactate concentration and a decline in ATP activity. Similar mitochondrial functional alterations can be observed in MELAS disease (mitochondrial myopathy, encephalopathy, lactic acidosis and stroke-like episodes) and MERRF syndrome (myoclonic epilepsy and ragged-red fiber syndrome) [112,113]. Taurine may prevent HF progression, reducing cardiotoxicity induced by detrimental activation of the sympathetic nervous system and angiotensin II [114]. Some studies in humans have demonstrated a beneficial effect of taurine supplementation in HF patients. They found that a few weeks of therapy improved exercise capacity, LVEF and NYHA class [115,116,117]. Consequently, in Japan taurine supplementation is approved for the treatment of HF. Nevertheless, to date, a clear association with mortality outcomes has not been demonstrated yet.

### 8.2. Carnitine

l-carnitine is a non-proteic, non-essential AA acting as a cofactor in fatty acid transport into mitochondria, acetyl-CoA production and glucose metabolism [118,119]. l-carnitine supplementation improves cardiac metabolic and left ventricular (LV) function and also has a protective effect on the ischemic myocardium, preventing fatty acid ester accumulation that occurs during acute ischemic episodes [120]. In HF patients, l-carnitine levels are reduced up to 50% [119]. The supplementation of proprionyl-l-carnitine to such patients has been largely studied, even in a double-blind placebo-controlled study. Overall, they demonstrated an improvement only in exercise capacity and in LV volumes [121,122,123,124]. According to Ferrari et al., these achievements occur without major modifications in the hemodynamic and neurohormonal state, but they are principally related to improved skeletal muscle metabolism [125,126]. To test the prognostic impact of carnitine supplementation in HF, Rizos conducted a double-blind placebo-controlled trial with 2 g/day vs. placebo administration and found an improvement in three-year survival [37].

### 8.3. Arginine

Arginine is a semi-essential AA involved in creatine synthesis. It is also the substrate of nitric oxide (NO) synthase which produces NO, leading to vasodilation and restoring endothelial function [127]. Some studies tested the role of arginine supplementation in HF. They found an improvement in endothelial-dependent vasodilation, muscular blood flow during exercise, functional capacity and exercise duration [128,129,130]. No data are available about survival outcomes in HF patients.

### 8.4. Mixtures of AAs

AA supplementation has been recently encouraged due to the possible beneficial effects on both the myocardium and skeletal muscle [112]. Aquilani et al. showed that 30-day oral administration of a mixture of 8 g/day AAs to elderly HF outpatients, in a double-blind fashion, improves exercise capacity measured by the cardiopulmonary exercise test (i.e., increased work, longer duration, more intense oxidative metabolism) and NYHA class. In addition, they found an improvement in post-exercise VO2 [131]. They concluded that AAs might positively affect circulatory function, muscle oxygen consumption, aerobic metabolism and recovery after exercise. Similar results have been obtained by Scognamiglio et al. and by our group after a three-month therapy with a wider mixture of AAs administered at 8 g/day [132,133]. Nevertheless, no studies have yet investigated their role in improving survival in HF patients.

## 9. Carnosine

l-carnosine is a dipeptide (β-alanine-L-histidine) present at high concentrations in the myocardium, skeletal muscles and brain [134]. It has anti-aging, antioxidant and immune-modulating properties, favoring NO production in the endothelial cells [135,136,137]. Recently, cardiometabolic proprieties of carnosine have raised an interest in clinical research [138]. Recent data suggest that carnosine supplementation may be effective in the prevention of type 2 diabetes in obese patients [139]. Our group has demonstrated that l-carnosine, added to conventional therapy, has beneficial effects on exercise performance and quality of life in stable, chronic HF [140]. Further data are necessary to evaluate its effects on LVEF and prognosis in chronic HF.

## 10. Multiple Micronutrient Supplementation

The approach of supplementing HF patients with multiple micronutrients is compelling. Theoretically, it may warrant the correction of several deficiencies, thus improving multiple metabolic pathways. Witte et al. analyzed the effect of a combination of 10 different micronutrients on LV function and quality of life in 30 elderly patients with stable systolic HF due to ischemic heart disease compared to controls [141]. This study suggested that a long-term multiple micronutrient supplementation can improve quality of life and LVEF in patients with HF [142]. A subsequent randomized, placebo-controlled study was conducted on 74 patients with chronic HF with a mixture of several micronutrients, including mainly vitamins and minerals (not AAs). Despite showing an increase of serum concentration of the supplemented micronutrients, no benefits in the change of LVEF, quality of life, natriuretic peptides and inflammatory marker levels were recorded [142]. In spite of the disappointing results of this study, further research is necessary to definitely clarify the best composition and the possible role of micronutrient supplementation in HF.

## 11. Conclusions

Micronutrients may act synergistically with traditional therapies in patients with HF, improving energetic metabolism and energy transfer. Despite plenty of small evidence, large clinical trials are still lacking. In addition, to date, only a few small studies have evaluated a multiple micronutrient approach to overcome the limitations with a single-supplement method. Future research is needed to address these important questions.

## Figures and Tables

**Table 1:** Selected studies on the prognostic role of plasmatic concentration of micronutrients in heart failure.

Supplement	First Author, Year	Patients	Mean/Median Plasmatic Concentration	Prevalence of Deficiency	Prognostic Impact
Coenzyme Q10	Molyneux, 2008 [31]	236 hospitalized for chronic heart failure (HF)	0.68 μmol/L (0.18–1.75)	-	Mortality: hazard ratio (HR) 2.0 at the ROC curve cut-point or 1.6 at the median value
	McMurray, 2010 [32]	1191 ischemic systolic HF	0.74 μg/mL (0.56–0.99)	-	Mortality: HR 1.5 at univariable analysis, not confirmed at multivariable one
25OH-vitamin D	Liu, 2011 [33]	548 HF	36.6 nmol/L (27.4–51.1)	75%	All-cause mortality and HF rehospitalization: HR 1.09 per 10 nmol/L decrease; all-cause mortality: 1.10 per 10 nmol/L decrease
Iron	Jankowska, 2010 [34]	546 stable systolic chronic HF patients	-	37% ± 4% (32% + 4% vs. 57% + 10% in subjects without vs. with anemia)	Death or heart transplantation: HR 1.58

**Table 2:** Selected trials about the prognostic effect of micronutrient supplementation in heart failure.

Supplement	First Author, Year	Type of Study	Patients’ Number	Dose	Duration	Survival Outcome	Results
Coenzyme Q10	Mortensen, 2014 [35]	Randomized controlled trial (RCT) (Q-SYMBIO trial)	420 (202 vs. 218)	100 mg × 3/die vs. placebo	106 weeks	Hospitalization for worsening heart failure, cardiovascular death, mechanical assist implantation, or urgent cardiac transplantation at two years	43% risk reduction
Vitamin D	Planned	RCT (EVITA trial, NCT01326650)	400	100 mcg/die vs. placebo	Three years	All-cause death at three years; cardiac transplantation, high urgent listing for cardiac transplantation, resuscitation, hospitalization, ventricular assist device implantation at three years	-
Iron	Jankowska, 2016 [36]	Meta-analysis	951 (509 vs. 342)	Variable vs. placebo	From five to 36 weeks	All-cause death or cardiovascular hospitalization; cardiovascular death or hospitalization for worsening heart failure	56% risk reduction; 61% risk reduction
l-Carnitine	Rizos, 2000 [37]	Open-label	80 (42 vs. 38)	2 g/die	Three years	Death at three years	3% vs. 18%

## Abbreviations

AAs	amino acids
ARBs	angiotensin receptor blockers
AHF	Acute heart failure
ATP	adenosine triphosphate
CoQ10	coenzyme Q10
GABA	γ-aminobutyric acid
HF	heart failure
HFrEF	heart failure with reduced ejection fraction
HFpEF	heart failure with preserved ejection fraction
NYHA	New York Heart Association
MACE	major adverse cardiovascular events
LVEF	left ventricular ejection fraction
NO	nitric oxide
TPP	thiamine pyrophosphate
6MWD	6-minute walking distance
